# Challenges and Solutions for Low-Temperature Lithium–Sulfur Batteries: A Review

**DOI:** 10.3390/ma16124359

**Published:** 2023-06-13

**Authors:** Yiming Liu, Tian Qin, Pengxian Wang, Menglei Yuan, Qiongguang Li, Shaojie Feng

**Affiliations:** 1 Queen Mary University of London Engineering School, Northwestern Polytechnical University, Xi’an 710129, China; liuyiming2206@mail.nwpu.edu.cn (Y.L.); tianqin@mail.nwpu.edu.cn (T.Q.); pxwang@mail.nwpu.edu.cn (P.W.); 2 State Key Laboratory of Solidification Processing, Center for Nano Energy Materials, School of Materials Science and Engineering, Northwestern Polytechnical University, Xi’an 710072, China; mlyuan@nwpu.edu.cn; 3 Anhui Province International Research Center on Advanced Building Materials, School of Materials and Chemical Engineering, Anhui Jianzhu University, Hefei 230601, China; fengshaojie@ahjzu.edu.cn; 4 Anhui Institute of Strategic Study on Carbon Dioxide Emissions Peak and Carbon Neutrality in Urban-Rural Development, Anhui Jianzhu University, Hefei 230601, China

**Keywords:** lithium–sulfur batteries, low temperatures, electrolyte, cathode, anode

## Abstract

The lithium–sulfur (Li-S) battery is considered to be one of the attractive candidates for breaking the limit of specific energy of lithium-ion batteries and has the potential to conquer the related energy storage market due to its advantages of low-cost, high-energy density, high theoretical specific energy, and environmental friendliness issues. However, the substantial decrease in the performance of Li-S batteries at low temperatures has presented a major barrier to extensive application. To this end, we have introduced the underlying mechanism of Li-S batteries in detail, and further concentrated on the challenges and progress of Li-S batteries working at low temperatures in this review. Additionally, the strategies to improve the low-temperature performance of Li-S batteries have also been summarized from the four perspectives, such as electrolyte, cathode, anode, and diaphragm. This review will provide a critical insight into enhancing the feasibility of Li-S batteries in low-temperature environments and facilitating their commercialization.

## 1. Introduction

In the context of the green and environmental protection society, the demand for rechargeable batteries is ever increasing, and the application range is also gradually becoming wider. Meanwhile, it is a significant challenge to break the theoretical bottleneck for commercial lithium-ion batteries (LIBs), consisting of a low-capacity anode and cathode.

Moreover, with the in-depth research of Goodenough et al. on lithium–sulfur (Li-S) batteries, such as the invention and development of key cathode materials for lithium-ion batteries, the potential development of lithium-ion batteries is being limited [1,2]. Li-S batteries have been widely considered because of their higher theoretical energy density, stronger environmental protection ability and lower cost, features with great promise when compared to alternative LIBs [3,4]. Unlike LIBs, working with the ion insertion mechanism [5,6], Li-S batteries are mainly based on the conversion reaction of active materials [7]. The active materials of the positive and negative electrodes are elemental sulfur and lithium metal, respectively, making the Li-S battery a high energy density, high performance, and low-cost sulfur cathode [8,9,10]. Therefore, Li-S batteries have an outstanding contribution to solving the problem of the subsequent development of the battery industry.

Unfortunately, Li-S batteries still face many challenges, especially at low temperatures. Li-S batteries, at low temperatures, suffer from the slow kinetics of cathode and anode reactions, which in turn leads to low capacity and poor cycle performance [11]. In addition, the shuttle effect of soluble lithium polysulfides (LiPSs, Li_2_S_x_, 2 < x ≤ 8), the insulation of S and insoluble sulfide (Li_2_S_2_ and Li_2_S) [12,13], the drastic volume change during charge and discharge, and the inherent dendrite growth of the lithium metal anode play a key role in the electrochemical performance [14].

In recent years, Li-S batteries have attracted extensive attention, but this is mainly focused on their performance at room temperature, including the idea of an interlaminar structure [15], designing novel electrode materials with enhanced electrochemical activity and stability [16,17], and optimizing the electrolyte composition and structure to improve ion transport and reduce the formation of lithium polysulfide [18]. However, there are still few systematic discussions on the operation of Li-S batteries at low temperatures.

**Figure 1:**
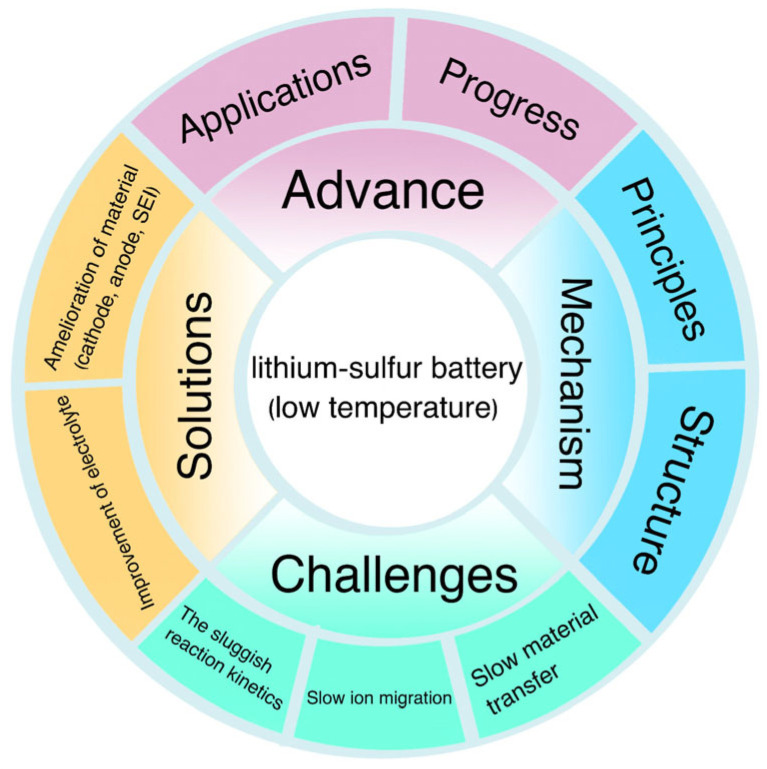
Schematic outline of major contents discussed in this review.

Research into Li-S batteries has been focused on solving several key problems and is moving towards commercialization [19]. However, new challenges may arise in terms of mass production and commercialization of the material [20]. This review focuses on the working mechanism and challenges faced by Li-S batteries at low temperatures and proposes potential solutions to overcome these challenges. The main failure mechanisms for low-temperature Li-S batteries have been discussed, as well as the advances and challenges for the anode, the cathode, and the electrolyte. Additionally, the perspectives and outlooks for low-temperature Li-S batteries have also been proposed (Figure 1). It is believed that this summary will facilitate the research of the low-temperature Li-S battery, which will eventually further promote their commercialization [21].

## 2. The Underlying Mechanisms of Li-S Batteries at Low Temperature

**Figure 2:**
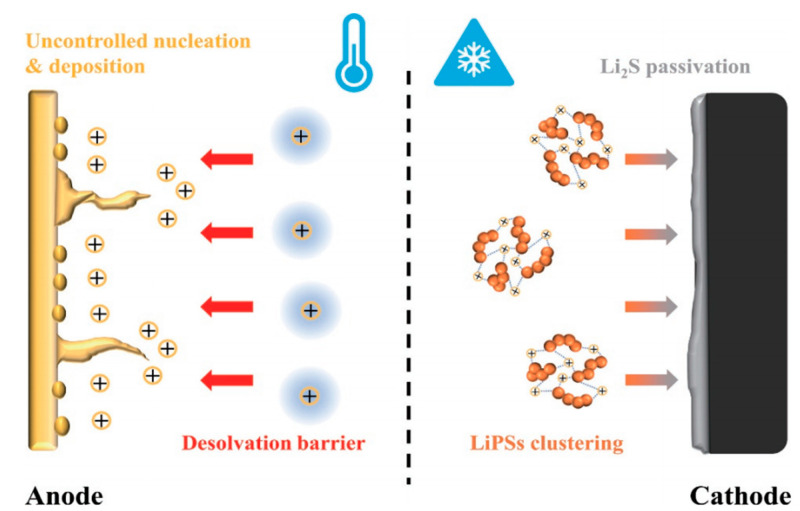
Illustration of Li-S battery failure mechanisms at low temperature [22].

The Li-S batteries are composed of a lithium anode, a sulfur cathode, an electrolyte, and a separator, suffering from the dilemma of reduced efficiency or even deactivation when they operate at low temperature. Specifically, the main failure mechanisms of Li-S batteries at low temperature include (i) a high Li ion desolvation energy barrier; (ii) uncontrolled nucleation and deposition of lithium; (iii) LiPSs cluster aggregation; and (iv) cathode passivation caused by Li_2_S film deposition (Figure 2) [22].

The high desolvation energy barrier of the Li ions at low temperature was determined to be the key factor leading to the failure of the battery. When the Li ions are bonded with the solvent molecule, the energy barrier for desolvation substantially increases at the electrochemical interface and further weakens local charge transfer capability [23]. To this end, the design strategies of solid electrolyte interphase (SEI) should be reconsidered to promote the dissociation of solvent molecules from the primary dissolved sheath of Li ions [24], which is favorable for the in-depth investigation of the high lithium ion dissolving energy barrier and would further enhance the performance of the lithium metal anode at low temperature.

The uncontrolled nucleation and deposition of lithium have an extremely negative effect on cycle performance. As the temperature decreases, the large-size deposited Li will evolve into dead lithium with an extensive surface, significantly reducing the Coulomb efficiency [25]. Accordingly, it is of great importance to develop effective strategies to mitigate lithium deposition and enhance the activity for Li-S batteries [26].

At low temperature, the low-discharge platform of Li-S batteries is firmly affected by the behavior of Li_2_S_4_ clusters [27]. In other words, the formation of Li_2_S_4_ clusters inhibit its conversion to Li_2_S, eventually leading to slow electrochemical reaction kinetics and capacity loss to the secondary voltage platform [28].

Regarding the cathode passivation by the film-like Li_2_S deposition, the nucleation size of Li_2_S shrinks with the decreased temperature, which leads to the prepassivation of the electrode surface and disappearance of three-phase interface of the active material, electronic conductor, and ionic conductor (electrolyte), thus terminating the electrochemical reaction. As a result, adjusting the deposition morphology of Li_2_S is of great significance to improve the capacity and reversibility of the positive electrode at low temperatures [29,30,31,32].

In fact, while the processes that limit the performance of low-temperature Li-S batteries are myriad, they all tend to have the same effect: capacity loss. Under a given voltage window, the reversible capacity of Li-S batteries is decreased with the decrease in temperature. For instance, below −20 °C, the reversible capacity of Li-S batteries is less than 25% of the primary capacity. After raising the temperature again to room temperature, the reversible capacity can be recovered as well. On the other hand, due to the irreversible deposition of lithium metal on the anode surface, capacity loss can also occur when the cell is charged at low temperature [33]. Both capacity degradation mechanisms for Li-S batteries at freezing temperature are mainly attributed to its increased internal resistance, which is caused by different physicochemical processes [34].

Optimizing the cell configurations can improve battery performance and address corresponding issues, which can be approached from various angles, including electrode and electrolyte optimization, and separator/interlayer design. The use of various 3D current collectors, such as porous current collectors, sandwich-type current collectors, and multilayered current collectors, can avoid the cracking. The utilization of binders on the cathode material can also enhance the contact performance between the coating and the electrode, and specific binders can improve specific properties of Li-S batteries. Zhou et al. introduced a binder, ammonium polyphosphate (APP), which not only facilitates the adhesion of electrode coatings but also utilizes its strong affinity with lithium polysulfides to hinder the diffusion and shuttle of polysulfide anions [35]. In terms of electrolyte, incorporating new types of electrolytes and using appropriate solvents and salts are commonly employed methods to improve its electrochemical performance. The addition of redox mediators (RMs) in the electrolyte is a highly promising approach for optimizing Li-S battery configurations, as RMs can regulate the oxidation reactions of sulfur [36]. Regarding the optimization of separators/interlayers, it can be categorized into three functions: (1) dealing with polysulfide diffusion; (2) improving the electrical conductivity for complete sulfur utilization; and (3) enhancing the kinetics of the conversion reactions of sulfur species. They, respectively, correspond to the key issues of polysulfide dissolution and the Shuttle effect, the insulating nature of sulfur species, and the poor kinetics in the scissoring of S-S bonding for Li-S batteries [37].

From the aforementioned perspectives, summarizing the challenges and solutions of Li-S batteries operated in low-temperature environments will provide critical insight into the development of new batteries.

## 3. Anode

Since the theoretical specific capacity of sulfur (1673 mAh/g) is much inferior to lithium (3860 mAh/g), the initial energy density is determined by the mass of sulfur in the whole system. Moreover, the instability at the surface and the bulk of the lithium anode during cycling seriously affect the capacity retention and cycle life [38]. According to recent progress, Li-S batteries are suffering from the following challenges and difficulties: (1) the Shuttle effect of Li ions [39]; (2) the uneven deposition of intermediate-term for SEI [40]; and (3) the serious dendrites and self-discharge of the lithium metal anode [4]. Among these factors, the serious dendrites of the lithium metal anode have the greatest impact on safety and stability [41]. The formation of lithium dendrites in lithium-ion batteries is attributed to the repeated deposition and stripping of lithium metal on the surface of electrodes during the charging and discharging process. Pore formation and surface defects can occur as a result of this process, leading to a high local potential that promotes the growth of dendrites [42]. Other factors such as temperature, current density, composition and concentration of electrolytes, electrode materials and structures, and the charging/discharging states of the battery can also contribute to dendrite formation. Lithium dendrites lead to serious safety issues, as they can cause short circuits, puncture the separator, and initiate uncontrolled reactions [43]. In order to overcome these difficulties and obtain stable lithium metal anodes, numerous strategies such as regulating the electrolyte, fabricating an artificial SEI layer, modifying the 3D current collector hosts and/or the lithophilic site, and exploring alternative anode materials have been employed [44]. However, research on Li-S batteries operating at low temperatures is still scarce. As a result, we mainly summarized and sorted some conventional methods and coping strategies under extreme conditions.

### 3.1. Exploration of Substitution and Improvement for Anode Materials

#### 3.1.1. Graphite

Graphite, with its flat intercalation potential, good capacity, and long cycle life, can effectively inhibit volume expansion and dendrite formation in lithium metal anodes. However, the related intrinsic drawbacks, such as poor Li diffusion kinetics in the interlayers, low intercalation potential, and relatively large interfacial resistance [45,46], usually result in the Li plating on graphite and restricting extensive applications in low-temperature conditions [45,46,47]. Due to the catalytic effect of the metal during the Li ions desolvation process, when mixed with 1 wt% of metal nanoparticles (Cu, Al, Sn), the light graphite oxide was able to deliver 30% of the theoretical capacity at −30 °C and 0.2 °C [48,49]. In comparison with the original graphite oxide electrode, the graphite oxide with coated and dispersed Sn provided a capacity of 152 and 94 m Ah g^−1^, respectively, at −30 °C. It was demonstrated that the low-temperature performance of graphite and LTO(Li_4_Ti_5_O_12_) anodes can be effectively improved by using copper nanoparticles on Super-P (Cu/Super-P) as a conductive additive [50,51]. Coating graphite with Al_2_O_3_ may also prevent Li deposition, improving its cryogenic performance [52].

The mild oxidation of graphite is expected to solve the problem of reducing the lithium overpotential of the graphite anode. Cao [53] and Wu et al. [54] innovatively utilized the heat treatment and concentrated nitric acid solution to lightly oxidize graphite, which exhibits better cycling performance at low temperatures. The reduced particle size and number of terminal unsaturated carbon atoms, forming nanoscale voids and channels as well as the formation of chemically bonded SEI, was attributed to this mild oxidation treatment [47].

**Figure 3:**
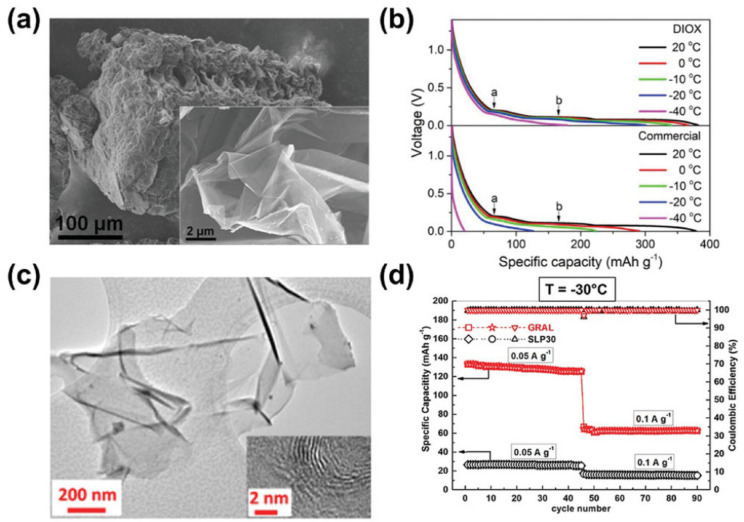
(**a**) Scanning electron microscopy (SEM) images of PGN−CNT and (**b**) its electrochemical performance at high rates and low temperatures with DIOX-based and commercial electrolytes [4]. (**c**) Transmission electron microscopy (TEM) images of GRAL anode and (**d**) the electrochemical comparison with SLP30 anode at −30 °C and 0.05/0.1 Ag^−1^ [56,57].

In addition, particle size reduction and structure modification can also improve the low-temperature performance of graphite [55,56]. The combination of thin graphite sheets with through-holes (porous graphite nanosheets, PGNs) and carbon nanotubes (CNTs) significantly shortens the diffusion path. The dominant mesopores and micropores in PGN-CNT anodes facilitate Li ion transport, resulting in a superior rate and performance at low temperatures (Figure 3a,b). After 500 cycles, the capacity retention was maintained at 90% for 0.75 m LiTFSI 1,3-dioxane (DIOX) electrolyte, and the reversible capacity was above 300 mAhg^−1^ at 0.1 °C and −20 °C. Ionic liquids were used to perform microwave exfoliation on expanded graphite and synthesized multilayer crystalline graphene (GRAL) (Figure 3c) [57]. The high surface area allows efficient electrochemical reactions, as evidenced by the 3–4 times greater capacity of GRAL compared to commercial graphite at −30 °C (Figure 3d). Moreover, oxided mesocarbon microbeads and expanded MCMB were prepared as well. The expanded MCMB with increased interlayer distance delivered 130 and 100 m Ah g^−1^ at −10 and −40 °C, respectively [58].

#### 3.1.2. Other Related Investigations

Recently, energy-dense Li-S batteries were achieved with the assistance of a gel polymer electrolyte, which was designed by redox chemistry between a Li_2_S cathode and Si anode. The rationally designed CoN@MCNF/Li_2_S cathode shows good temperature adaptability at around −20 °C [59].

### 3.2. Electrolyte Chemistry Modulation

Jiao et al. [60] systematically studied the performance of lithium metal anodes over a range of temperatures. A variety of electrolyte systems have been studied, such as a cosolvent of 1,3-dioxolane (DOL)/1,2-dimethoxyethane (DME), and a cosolvent of ethylene carbonate (EC) and dimethyl carbonate (DMC). Both drupe-like and small spherical deposits of lithium were detected at high temperatures of 60 °C. It was shown that between five degrees and minus fifteen degrees, there will be branching of lithium metal anodes. However, at higher temperatures, the C–C and C–O groups of organics in the SEI are enriched, resulting in a low modulus, flexible SEI structure. In thermodynamic and kinetically favored depositional conditions, low-current density, high Coulombic efficiency as well as a nondendritic structure has been achieved in these electrolytes.

**Figure 4:**
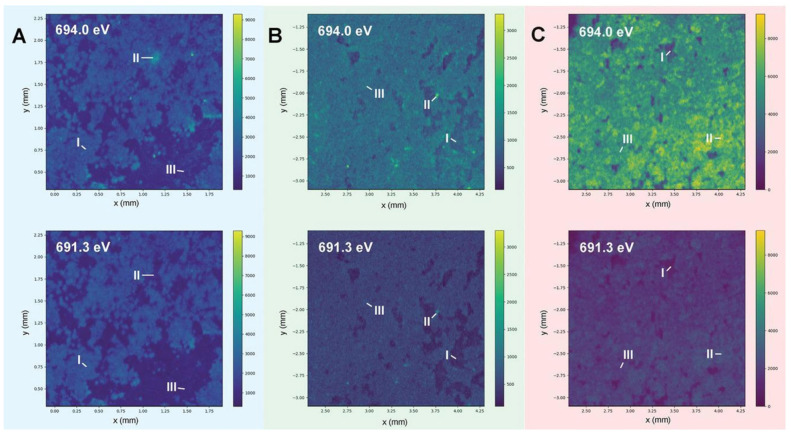
Investigations on temperature effects on the SEI structure using advanced techniques. (**A**–**C**) Displays the synchrotron energy-dependent XRF mappings of the cycled lithium metal anode at 0, 25, and 60 °C, respectively [61].

Compared with low temperatures, conventional carbonate LiFP_6_ electrolytes are preferred over poorer SEI layers, with lower Coulombic efficiency and shorter cycle life at high temperature [61]. At 0 °C, a homogenous SEI layer was formed that contained uniformly distributed F-containing species, displaying an extended cycle lifetime and increased Coulombic efficiency (Figure 4). The XRF mappings and micro-XANES images illustrate that F and O are inhomogeneously distributed over most of the surface and from the surface to the bulk, which is important to understand. In particular, when the cycle temperature reaches below 60 °C, the internal composition of the SEI is mainly composed of inorganic Li_2_CO_3_, and the surface composition is mainly composed of organic oxygen and LiF. These species are produced by the reaction between the electrolyte and the deposited lithium, thus causing rapid failure when building a thick SEI.

Robert [62] and co-workers demonstrated that using the positive temperature gradient method (the positive electrode is set to 40 °C and the negative electrode is set to 0 °C) could produce uniform lithium quiescence and reduce deleterious side reactions. In typical LiTFSI-LiNO_3_-based ether electrolytes, after one hundred cycles, the symmetrical cell with positive thermal gradient exhibited the lowest voltage hysteresis and the longest cycle life. Whereas, under negative temperature gradient conditions (negative pole set to 0 °C and positive pole set to 40 °C), a rapid degradation of battery performance was monitored after only 15 cycles. It demonstrated that the very first lithium seeding state enabled control of quality and cycle life of Li metal anodes. In particular, negative temperature gradients can lead to cold seeding and thermal stripping processes, which generally produce inhomogeneous coatings with high aspect ratios, whereas positive temperature gradients can produce uniform initial lithium quiescence with good long-term cycling performance. These external temperature gradient methods are conducive to stabilizing Li metal anodes.

To achieve an ultra-low temperature lithium metal anode, Xia [63] and co-workers developed a cosolvent electrolyte system by adding electrochemically “inert” dichloromethane (DCM) as diluent to a concentrated ethyl acetate (EA)-based electrolyte. By adding DCM diluent, the solvated structure was changed. In EA solution, the mobile DCM molecules wrap the salt clusters of LiTFSI. Due to this unique structure, the electrolyte exhibits high ionic conductivity, low viscosity, and low-temperature performance. Although the stripping/plating performance of the Li metal anode was not shown, the Li//PI battery exhibited good cycling performance at −70 °C and good performance at low temperatures. However, based on the investigation of the Golmohammad group, the newly fabricated solid-state electrolytes show good stripping/plating performance toward the metallic Li anode under such conditions [64,65,66].

### 3.3. Artificial SEI Layer

**Figure 5:**
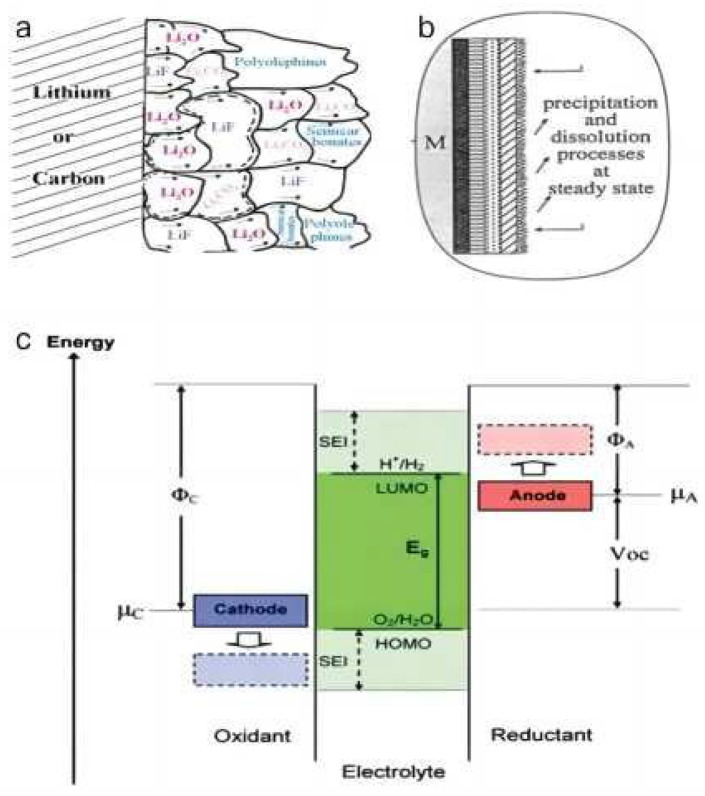
Schematic illustration of the proposed (**a**) mosaic structure [53], (**b**) multilayered structure [70] of the SEI. (**c**) Schematic open-circuit energy diagram of battery electrolyte. Φ_A_ and Φ_C_ are the anode and cathode work functions. E_g_ is the window of the electrolyte for thermodynamic stability. A µA > LUMO and/or a µC < HOMO requires a kinetic stability for the formation of an SEI layer [71].

The problem of the high lithium ion desolubilization energy barrier at low temperatures can be effectively mitigated by artificially adjusting the SEI film. The slow process at the lithium anode occurs when the solvated Li attempts to enter the anode structure, where it must peel off its primary solvated sheath [67]. This dynamically challenging process, often referred to as the “charge transfer” component due to its characteristic 50–70 kJ mol^−1^ activation energy barrier, is the fundamental reason why LIBs cannot be charged at low temperatures. However, the activation energy of the Li ions’ interfacial transfer is different in the presence of different surface films (SEI), and the dynamics of cathode–interface Li ion transfer are also affected by the composition of SEI film [68,69]. Therefore, the artificially modified SEI film effectively affects the “charge transfer” process, providing a beneficial strategy to solve the high Li ion desolubility barrier problem (Figure 5).

### 3.4. The 3D Current Collector Hosts and/or Lithophilic Site Modification

The 3D current collector hosts and lithophile site modification are effective to achieve high-performance lithium anodes and fast conversion kinetics [45]; their feasibility and reliability in extreme hot and cold conditions, however, have been studied relatively infrequently. Peng and co-workers [72] used chemical vapor deposition to produce graphite-coated Ni-Fe foam (graphite @Ni-Fe). It exhibited good cycle performance, and the capacity retention rate was 98.10% after 100 cycles at −50 °C and 0.4 A cm^−2^, with a Coulomb efficiency over 98.3%. At present, zinc batteries are performing at low temperature, but lithium batteries are not.

## 4. Cathode

### 4.1. Challenges

At room temperature, the reaction of LiPSs species equilibrates rapidly. The reaction consists of two main processes called ”high” and “low” discharge plateaus in the discharge voltage profile [44]. On the contrary, the two plateaus cannot be observed anymore and multistep processes take the place of the two plateaus in the reaction at low temperature, manifesting the slackening of the LiPSs’ equilibration and sluggish kinetics [73].

**Figure 6:**
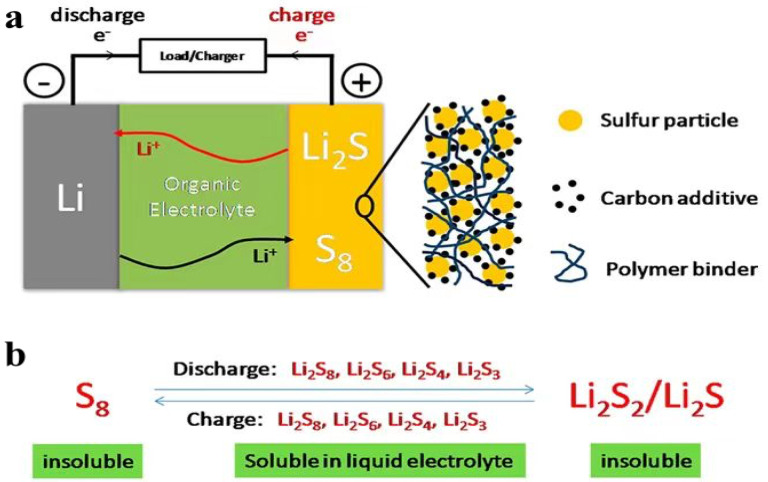
(**a**) Illustration of the charge/discharge process involved in rechargeable Li−S batteries consisting of lithium metal anode, organic electrolyte, and sulfur composite cathode and (**b**) charge/discharge process involving the formation of soluble lithium polysulfides (Li_2_S_8_, Li_2_S_6_, Li_2_S_4_, and Li_2_S_3_) and insoluble Li_2_S_2_/Li_2_S [79].

Moreover, the Li-S battery generally suffers from capacity failure at low temperatures. A complex solid–liquid–solid phase transition occurs under the discharge process. In the first section, the cyclo-S_8_ with ring structure is broken and forms Li_2_S_8_ through a solid–liquid two-phase reaction [74]. Regarding the second section, Li_2_S_8_ reduces to insoluble and high-crystallized Li_2_S_2_/Li_2_S [75]. Compared to the first section, it possesses higher reaction potential and more rapid kinetics [40]. The reaction kinetic is suppressed in the second section, while Li_2_S_2_ (the low-order intermediate of the reaction) has a tendency to assemble in the electrolyte, finally forming highly crystalline particles and suppressing the reaction at the second voltage platform [27,28]. In addition, the second section counts for about 50% theoretical capacity due to which the second step not only reduces the reaction rate but also causes severe capacity failure. Consequently, the normal commercial Li-S batteries can only deliver 20% capacity compared to their room-temperature state [76]. Several studies have shown that sulfur host materials with catalytic activity can enhance the reaction rate of the second step and increase the capacity at low temperature; however, the enhanced capacity still lags behind that of room temperature (Figure 6) [40,77,78].

### 4.2. Advances

**Figure 7:**
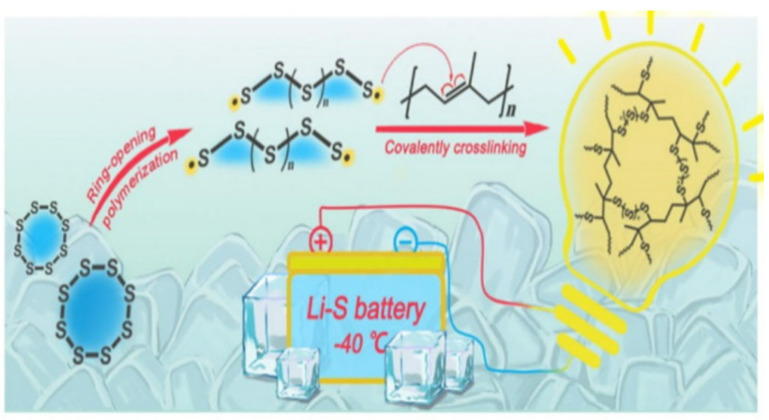
Schematic diagram of the synthesis of SRVCR [40].

Wang et al. managed the ring-opening reaction of S_8_ and its subsequent reaction with cis-polyisoprene (RUB). They obtained a novel cathode organosulfur material, sulfur-rich vulcanized clay rubber (SRVCR), from the reactions. The SRVCR material exhibited a high capacity of up to 560 mA hg^−1^ at 1 °C and at the low temperature of −40 °C. In addition, it also exhibits a low-capacity failure rate of 0.022% per cycle after 300 cycles at room temperature (Figure 7) [40]. It is noteworthy that multiwalled carbon nanotubes (MWNTs) have been employed by the thermal decomposition of CH_4_ to solve the polysulfide sediment problem. The treated MWNTs served as an inactive additive towards the positive electrodes for Li-S batteries, accelerating the polysulfide adsorption and further enhancing the cycle life and the rate capacity of the sulfur cathode [80].

**Figure 8:**
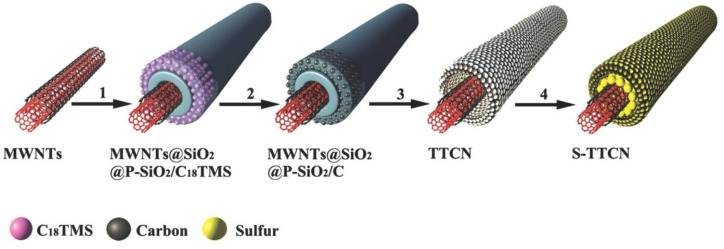
Schematic illustration for the formation of S-TTCN composite [85].

Tube-in-tube carbon nanostructure (TTCN) was utilized as cathode material for Li-S batteries, which can also remarkably improve rate performance (Figure 8) [81]. Similarly, a carbon sphere has been designed to serve as host material, ensuring enough space for sulfur storage and electrolyte accessibility. It showed outstanding cycle performance, rate capacity, and a low capacity degradation of about 0.1% per cycle after 100 cycles [82]. An integrated cathode (G/CNT-S//G/CNT), comprising condensed graphene/carbon nanotube (G/CNT) aerogels, was reported, which features a large surface area of 363 m^−2^ g^−1^, a huge mass density of 1.64 g cm^−3^, a high conductivity of 67 S m^−1^, and a superior electron-ion transport channel. Due to these striking features, the cathode delivered a high reversible capacity of 1286 mA hg^−1^ at 0.2 °C and exhibited a low failure rate of 0.06% per cycle at 2 °C after 500 cycles. Furthermore, this cathode also achieved the high volumetric capacity of 1841 Ah L^−1^ and volumetric energy density of 2482 Wh L^−1^ [83].

Recently, much endeavor has been paid into the advanced hollow structure of sulfur [84,85]. A unique and hollow sulfur nanostructure had been designed, which contributes a large inner space to bear the high sulfur load. Moreover, the hollow structure has enough space to adapt to the swell during the lithiation process and promises high flexibility for Li-S batteries. The integrated shell can lessen the solubility of LiPSs as well. Consequently, the hollow sulfur nanostructure can significantly enhance the performance of Li-S batteries [86]. It is of great significance in the development of cathodes for Li-S batteries, especially at low temperatures.

Zhang et al. successfully synthesized a novel graphene oxide-Zn (II)-triazole complex, which displayed extraordinary performance at the low temperature of −20 °C. As a consequence of the import of metal ions (Zn(II)), amine groups and the N penta-heterocycle, the fabricated cathode material delivered a reversible capacity of 315 mAh g^−1^ (0.5 °C) over 100 cycles at −20 °C [87]. Moreover, Gao’s group designed a free-standing host implanted with an effective CoFe catalyst for the polysulfide translation, which can reach a brilliant battery capacity of 836 mAh g^−1^ at 0.2 °C and an excellent cycle stability of 94.5% capacity retention rate after 100 cycles at −20 °C [88]. A nitrogen-enriched carbon/sulfur composite cathode has been designed, achieving strengthened polysulfide immobilization and retarded capacity failure [89]. In addition, a graphene-supported BN composite was prepared and enabled Li-S batteries to behave excellently at low temperature [78]. Additionally, the Li_3_V_2_(PO_4_)_3_ cathode can deliver 67% battery capacity at the low temperature of −40 °C [90]. Furthermore, Akridge et al. designed a novel cathode material for an Li-S battery operated even at −60 °C, exhibiting a high energy density of 250 W h/kg [83,91].

### 4.3. Outlooks

The Li-S battery cathode at low temperature mainly suffers from low kinetics and capacity failure, attributed to the sediment of polysulfide. Additionally, the physical approach is considered one of the most efficient methods to mitigate the aforementioned challenges. Owing to the failure of nonpolar C-C bonds, ever-increasing efforts towards the creation of hollow structures has been made to boost the electrochemical performance, which are beneficial to accelerate the commercialization of Li-S batteries.

## 5. Electrolyte

### 5.1. Challenges

The electrolyte of x′Li-S batteries faces severe challenges at low temperatures as well. Both main components of the electrolyte feature a low freezing point below −50 °C. In comparison with room temperature, the electrolyte suffers from rapidly increasing viscosity, leading to sluggish kinetics, at low temperatures [92,93]. On the other hand, the electrolyte faces the dissolution of sulfur, causing irreversible loss of the active content of the sulfur cathode. Electrolyte exhaustion also troubles the Li-S battery. Due to the highly active species dissolution and polysulfide anion erosion, the electrolyte consumption and polysulfide accumulation are often severe, which is mainly attributed to the low capacity and poor cycle life [94]. Herein, it is urgent to develop and explore novel electrolyte systems to solve/inhibit the above problems.

**Figure 9:**
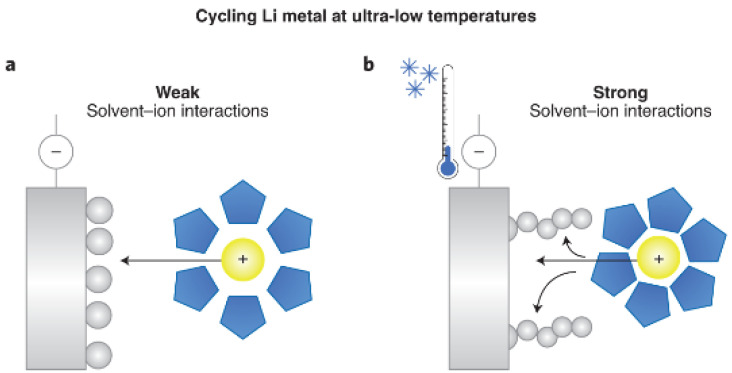
(**a**) The weak interaction between the solvent and lithium ions at low temperatures led to fast desolvation and uniform electrodeposition. (**b**) The solvent–ion interactions in conventional electrolytes are strong, leading to sluggish desolvation and dendritic lithium deposition morphologies [23].

Due to the complex characteristics of Li-S batteries and severe condition, the high performance of Li-S batteries was impeded [94,95]. The solubility and accumulation of LiPSs limit the electrochemical reactions and, thus, the capacity for Li-S batteries [96]. Moreover, the nucleation of Li is another challenge of Li-S batteries. The discharge product, Li_2_S, would nucleate and swiftly accumulate at low temperatures. The insulated Li_2_S can introduce a passivation layer on the electrode, which may impede the cathode reaction and ultimately result in sulfur consumption. Furthermore, the accumulation of Li_2_S obstructs the pores of the sulfur host and electrode, blocking ion diffusion and leading to the growth of Li_2_S [97]. At low temperatures, in Li desolvation, the strength of the bond between Li and the solvent molecules is usually considered to be the main cause of impedance (Figure 9) [23]. Consequently, researchers are focusing on reducing the proportion of solvent-to-anion in the Li solvation sheath or weakening the bond strength between Li and the dipole. Nevertheless, efforts to increase the anion or employ nonpolar diluents have proven insufficient in improving the performance. Additionally, the main problems are reducing the robust coordination intersection between Li and polar functional groups of solvents. Therefore, to reduce the intrinsic solvating power of solvents, an important strategy is the deliberate design of polar solvents with substituted fluorine atoms. Due to their high electronegativity and low polarity, such atoms are promising for rational solvent design [98]. In addition, cosolvent electrolytes were employed to ameliorate ion transportation, leading to the interface resistance between anode and electrolyte increasing, generally leading to irreversible plating at low temperatures [23]. To solve the problem mentioned above, the development of high-performance electrolytes remains a critical challenge.

### 5.2. Advances

Importantly, the amelioration of electrolytes for Li-S batteries has been widely explored, including solvent selection, lithium salt optimization, and additive participation.

#### 5.2.1. Solvent Selection

The common electrolytes of Li batteries are predominantly composed of a combination of two or more solvents mixed with one or more lithium salts to meet the conflicting requirements. Consequently, multiple solvents with distinct physical and chemical properties are integrated to address multiple functions simultaneously [92]. The choice of solvents is determined by factors including their compatibility with other components, capacity to dissolve the lithium salt, and stability towards electrochemical reactions.

Huang et al. proposed the 1M LiPF-FEC/AN (fluoroethylene carbonate/acetonitrile) electrolyte with varying solvent ratios to investigate the impact of the FEC/AN ratio on the electrochemical performance. It was revealed that the electrochemical performance is dependent on the FEC/AN ratio, which allowed them to distinguish between the interfacial chemistry and the bulk properties of the electrolyte. The optimized electrolyte, consisting of 1 M LiPF6-7FEC/3AN, enabled exceptional high-rate capability, attributed to the interfacial chemistry of the Li-FEC-AN complex. To quantify the impact of the Li-FEC-AN complex and interfacial chemistry, the Li coordination environment in 7FEC/3AN was analyzed. For the film-forming ability, the Li solvation sheath required dominance by FEC, with the AN/FEC coordination number ratio averaging ≤0.26. Regarding ionic conductivity, AN significantly reduced the Li diffusion time among solvation configurations to 77 ps, which was a full order of magnitude lower than with the 1 M LiPF6-FEC electrolyte [99].

Zhang et al. delved into the potential of N-methyl pyrrolidone (NMP), a newly introduced cosolvent, in PC-based electrolytes. Solvation chemistry analyses and in situ characterizations were employed to provide insight into interfacial chemistry from different perspectives. Based on these results and theoretical calculations, it was proposed that N-methyl pyrrolidone can mediate the reduction process of propylene carbonate, which, in turn, facilitates the SEI layer formation [100].

Due to the poor cycle ability and the potential hazard of PC, ethers have attracted extensive attention. Mikhaylik et al. reported a novel ratio of electrolytes of 40% 1,3-dioxolane (DOL) and 86% dimethoxyethane (DME), showing better performance at the low temperature of −1 °C [101]. To further elevate the electrochemical performance, both DEE and DOL/DME were used. It was revealed that the 1 M LiFSI DEE showed a great performance at the low temperatures of −23 °C, −40 °C, and −60 °C, which provides an extensive strategy for both further research and commercialization [102].

#### 5.2.2. Lithium Salt Optimization

Lithium salt is an essential component for the electrolyte of Li-S batteries that plays a significant role in regulating their low-temperature performance [103]. To improve the low-temperature performance, it is necessary to explore appropriate lithium salt with compatibility with the solvent and desired properties, enabling Li-S batteries to operate effectively in a range of temperatures and provide reliable performance over their lifespan.

**Figure 10:**
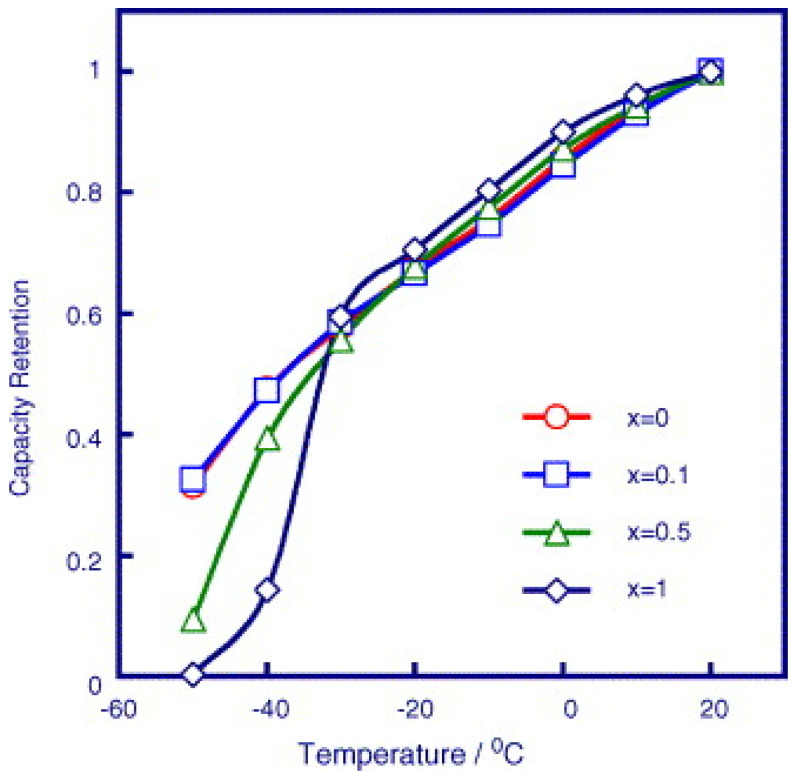
Effect of salt ratio on the low temperature performance of Li/LiFePO_4_ cells with an electrolyte of 1.0 m (1 − x)LiBF(4 − x)LiBOB 1:1:3 PC/EC/EMC (0.8 m for x = 1), in which the cells were cycled at 1 °C and the capacity retention is defined as the ratio of discharge capacity at a specific temperature to the capacity at 20 °C [104].

It was found that LiBF_4_ has an excellent performance at low temperatures while it fails to perform well at high temperatures. Therefore, LiBF_4_ was combined with Lithium bis(oxalate)borate (LiBOB), an electrolyte having the opposite character as LiBF_4_. A Li/LiFePO_4_ cell with 90:10 LiBF_4_-LiBOB salt can provide up to ~30% capacity at ~3.0 V even at the low temperature of −40 °C (Figure 10) [104]. Lin et al. proposed an electrolyte for Li||Ni_0.5_Co_0.2_Mn_0.3_O_2_, exhibiting brilliant capacity (50% capacity at room temperature) at the low temperature of −40 °C [105]. With respect to polysulfide dissolution in electrolytes, A simple electrolyte, LiFSI in dimethoxyethane, has been developed, displaying a stable cycle performance with an attenuation rate less than 0.013% per cycle [106]. It is recommended to choose lithium salt for novel electrolytes. A flame-retardant concentrated electrolyte (6.5 M LiTFSI/FEC) was employed to prevent the nucleation of Li_2_S, showing good performance at the low temperature of −10 °C [107]. All in all, the choice of lithium salt can be determined by the requirements of Li-S batteries and has a vital effect on the performance of Li-S batteries.

#### 5.2.3. Additives

**Figure 11:**
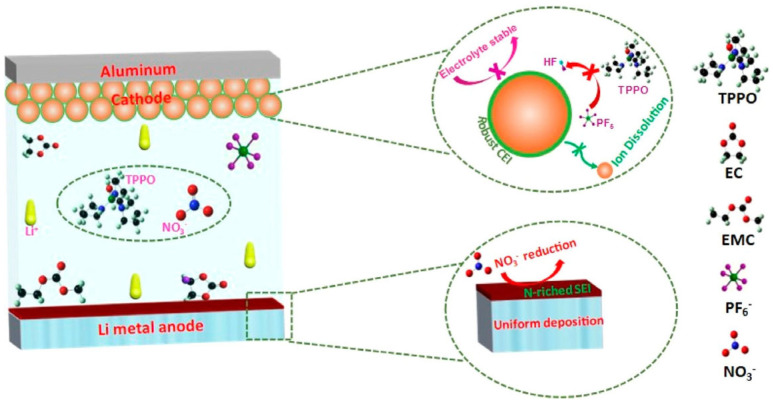
Illustration of interfacial chemistry at cathode−electrolyte and anode−electrolyte interfaces [110].

Additives, as a significant component for the electrolyte, can contribute to the electrochemical performance for Li-S batteries. LiNO_3_ is a popular additive due to its excellent character of dissolving the lithium deposit. Xiao et al. employed LiNO_3_ as additive for Li-S batteries, showing high Coulombic efficiency over 95% [108]. Liu et al. found that the additive LiNO_3_ can reduce the nucleation overpotential and achieve excellent cycle stability over 300 h [109]. Other additives can also ameliorate the electrolyte. Xiao et al. innovatively used tris(pyrrolidinophosphine) oxide (TPPO) as an additive. They discovered that the TPPO can provide a strong interaction with lithium, making the LiNO_3_ easily dissolve in the electrolyte and efficiently preventing the Li nucleation. Due to its exceptional role, the Li/LiFePO_4_ with TPPO still performed a stable performance when tested for stability at the low temperature of −15 °C (Figure 11) [110]. Pyrene was employed to boost the charge transfer process. Compared to the electrolyte without pyrene, the one with pyrene enhanced 25.1% capacity at a low temperature of −15 °C after 50 cycles [111]. Considering the poor solubility of LiNO_3_ in commercial carbonate electrolytes, LiNO_3_ and TMU were both adopted to boost the performance by forming conductive and stable SEI and suppressing HF generation. It achieved 96.14% capacity at a current density of 1 mA cm^−2^ and the low temperature of −15 °C [112].

In summary, as a vital part of Li-S batteries, the electrolyte modification generally suffers from great challenges, and extensive attention has been attracted to improve their performance. It mainly focuses on the problems of rapidly increasing viscosity, the dissolution of sulfur and LiPSs, the shuttle effect, the nucleation process of Li, and the strength of the bond between Li and solvent molecules. Additionally, more efforts should be turned towards these aspects, thus accelerating its commercial application.

## 6. Conclusions and Outlook

In short, the advances for low-temperature Li-S batteries have been reviewed, and the challenges have also been proposed: (1) the wettability and ionic conductivity decrease in the electrolyte; (2) the sluggish interfacial reaction kinetics, attributed to the high Li^+^ desolvation energy, sluggish charge transfer, and Li ion diffusion through the SEI layer; (3) the uncontrolled lithium nucleation and deposition and low Li^+^ diffusion rate; (4) the cathode passivation caused by aggregation of LiPSs and Li_2_S; and (5) Li plating on the surface of the anode, contributing to the short circuits.

To overcome these challenges, a few implementable strategies are proposed: (1) rational tailoring of solvents, lithium salts, and additives to boost low-temperature ionic conductivities, reduce desolvation energy, and form thin, inorganic-rich SEI layers; (2) doping, surface coating, as well as structural design for electrode, etc., to stimulate Li^+^ diffusion; (3) suppressing Li seeding and dendrites; and (4) other strategies, such as self-heating and/or optimization of battery configuration.

Currently, research on low-temperature Li-S batteries has made significant progress and laid the groundwork for future research. There is, however, still a considerable gap in practical application. Additionally, the advanced prospects for the development of low-temperature Li-S batteries have been proposed:

1. Pay more attention to the performance of the low-temperature Li-S batteries with high loading. In the current research, a considerable electrochemical performance has been achieved under a relatively low mass loading. However, it is inevitably necessary to enhance the mass loading when the Li-S batteries are for commercial application.
2. Construct an artificial SEI layer towards cryogenic applications. As ideal SEI layers are difficult to achieve at low temperatures, it may be an effective and reasonable strategy to pretreat the lithium surface at room temperature, constructing artificial SEIs and, thus, improving low-temperature performance prior to low-temperature operation.
3. Further investigate the mechanism for low-temperature Li-S batteries. In contrast to the transformation process of LiPSs at room temperature, aggregation and slow ion transportation at low temperatures can affect the kinetics of sulfur species. It is, therefore, suggested that experimental investigation and theoretical simulation should be combined for future studies. It is of significant importance to explore the critical rate-limiting steps in the transformation of LiPSs at low temperatures.
4. Evaluate the pouch-cell performance. Li-S batteries are gradually being developed from basic research to practical applications. To bridge the gap between laboratory experiments and industrial production, there is a growing body of work to verify the feasibility of the proposed strategies in practical applications. Consequently, in future research and development of low-temperature Li-S batteries, pouch cells should also be fully utilized to assess battery performance.

As the requirements for flexible batteries, such as the foldable smartphones from Huawei and Samsung, in the current market continue to increase, there is a growing interest in wearable electronic devices [113]. Li-S batteries have become a choice for flexible battery due to their low cost and high energy density, making them suitable for the aforementioned electronic devices [114,115]. In response to global warming and carbon emission issues, hybrid electric vehicles have been introduced to the market and attracted extensive attention, providing an application market for Li-S batteries. Due to their tremendous research and development potential and excellent cost–performance ratio, Li-S batteries have become one of the choices in the battery market [116]. Sion Power Corporation has already developed batteries with an energy density of 350 Wh kg^−1^. Their batteries have shown excellent results in unmanned aerial vehicle applications [117]. These results demonstrate the promising future of Li-S batteries in the electric vehicle industry.

## Data Availability

The authors do not have permission to share data.
